# The efficacy and safety of neoadjuvant treatment with the PD-1 inhibitor for locally advanced colorectal cancer: a meta-analysis

**DOI:** 10.3389/fonc.2024.1416943

**Published:** 2024-12-02

**Authors:** Yan Yu, Lin Huang, Rong Yan, Min Jiang, Shuang-Jiao Li, Wang-Dong Fan

**Affiliations:** ^1^ West China School of Public Health, Sichuan University, Chengdu, Sichuan, China; ^2^ West China Fourth Hospital, Sichuan University, Chengdu, Sichuan, China

**Keywords:** programmed cell death protein-1, PD-1, locally advanced colorectal cancer, neoadjuvant therapy, meta-analysis

## Abstract

**Objective:**

To systematically evaluate the efficacy and safety of PD-1 inhibitors in neoadjuvant therapy for locally advanced colorectal cancer (LACRC).

**Method:**

Retrieved from PubMed, Embase, and the Cochrane Library, all relevant studies about PD-1 inhibitors for neoadjuvant treatment of LACRC were collected from inception to 31 December 2023. The efficacy was assessed by the rate of pathological complete response (PCR), clinical complete response (CCR), and major pathological response (MPR), and the safety was evaluated by the incidence of all adverse effects (TRAEs). Subgroup analysis was conducted by experimental design, types of PD-1 inhibitors, and disease types.

**Result:**

A total of 803 patients were included in 21 studies. The results of the meta-analysis showed that the PCR rate of PD-1 inhibitors in the treatment of LACRC was 54% (95% CI: 43%–65%, P<0.05); the CCR of anti-PD-1 was 40% (95% CI: 26%–54%, P<0.05); the MPR was 66% (95% CI: 56%–76%, P<0.05); and the irAEs was 27% (95% CI: 17%–37%, P<0.05). Subgroup analysis showed that the PCRs in prospective studies and retrospective studies were 49% (95% CI: 32%–66%, P<0.05) and 57% (95% CI: 42%–73%, P<0.05), respectively. Among the 803 patients, 619 (77%) were diagnosed with rectal cancer (RC), and the PCR and MPR were 49% and 65%, respectively; 184 (23%) were diagnosed with colorectal cancer (CRC), and the PCR and MPR were both 67%. In our meta-analysis, types of PD-1 inhibitors, including sintilimab, toripalimab, camrelizumab, avelumab, pembrolizumab, and tislelizumab, and patients who received PD-1 inhibitors alone or in combination achieved good PCR rates.

**Conclusion:**

Neoadjuvant therapy combined with a PD-1 inhibitor has a favorable PCR and relatively low incidences of irAEs for patients with LACRC, suggesting that this regimen including a PD-1 inhibitor is significantly effective and sufficiently safe.

## Background

Colorectal cancer (CRC) is one of the most common malignant gastrointestinal cancers, and it ranks third in incidence and second in mortality worldwide among all cancers (1). CRC has an annual incidence of approximately 732,000 cases worldwide (2). Based on statistical data, the annual global mortality rate from the disease exceeds 800,000 individuals (3). The CRC poses a serious health threat to residents because CRC is a highly heterogeneous disease and not easy to detect; in addition, approximately 60% of patients with CRC have locally advanced diseases upon diagnosis (4), which accounts for a large proportion of rectal cancers.

The current standard treatment for patients with LACRC involves neoadjuvant therapy followed by total mesorectal excision, with the option of adjuvant chemotherapy (5). Neoadjuvant therapy has been shown to enhance prognosis by reducing local recurrence, inducing tumor regression, downstaging clinical presentation, and increasing the rate of sphincter preservation. However, neoadjuvant chemotherapy can cause postsurgical issues like anastomotic leakage, poor perineal wound healing, long-term problems such as urination and sexual dysfunction, and loss of anal sphincter function (6). Recently, immune checkpoint inhibitors (ICIs), including PD-1 inhibitors, have transformed cancer treatment with their superior efficacy. They are particularly effective for treating most microsatellite instability-high (MSI-H) and mismatch repair-deficient (dMMR) CRC (7). Several studies have demonstrated the efficacy of neoadjuvant therapy with PD-1 inhibitors, but with a small sample size (8–10). However, a comprehensive analysis of the efficacy and safety of neoadjuvant immunotherapy for patients with LACRC remains limited. Consequently, an extensive review of relevant literature was undertaken, succeeded by a meta-analysis, to rigorously evaluate the efficacy and safety of neoadjuvant immunotherapy utilizing PD-1 inhibitors for LACRC.

## Methods

This systematic review and meta-analysis adhered to the reporting guidelines outlined by the Preferred Reporting Items for Systematic Reviews and Meta-analyses (PRISMA) (11).

### Search strategy and selection process

A systematic literature search of Embase, PubMed, and the Cochrane Library was conducted from the time when the database was established until 31 December 2023, limited to the English language. The key terms included “Neoadjuvant therapy,” “Colon Cancer,” “Colorectal Cancer,” “Rectal Cancer,” “PD-1 Inhibitors,” “Programmed cell death protein one inhibitor,” and “Immune checkpoint inhibitor”. We also searched the references to the literature that had been retrieved. Two reviewers independently reviewed the title and abstract of all papers and a full-text review of potentially eligible studies. A third reviewer adjudicated any discrepancies or conflicts.

### Inclusion and exclusion criteria

#### Inclusion criteria

1. Patients diagnosed with LACRC pathologically
2. Patients were administered PD-1 inhibitor therapy in conjunction with neoadjuvant treatment for LACRC
3. No history of ICIs or other experimental drug therapy
4. The literature provides the outcome indicators, including pathological complete response (PCR), clinical complete response (CCR), major pathological response (MPR), and incidence of all adverse effects (TRAEs), which can be extracted or calculated from the original research
6. Study design: randomized controlled trials, case–control studies, cohort studies

#### Exclusion criteria

1. Studies published as reviews, letters, case reports, and duplicate literature
2. Studies that did not report relevant outcome measures or for which relevant data were not available
3. The study is based on cell or animal experiments
4. Patients with other tumors

### Data extraction

Two authors independently carried out the screening and extraction processes. A third reviewer adjudicated any discrepancies or conflicts. For the lack of information, we contacted the original author as much as possible. The following data elements were extracted from the included studies, including the first author, publication year, disease type, the number of patients, gender, age, the clinical stage, study design, types of research, treatment cycle, types of PD-1 inhibitors, median follow-up period, and the outcome measures data. The PCR/CCR/MPR evaluated the efficacy, and the adverse effects measured the safety.

### Quality evaluation

The quality of included RCTs was evaluated using the Rob risk of bias quality assessment (12), and non-RCTs were carried out using the Newcastle–Ottawa Scale(NOS) (13).

### Statistical analysis

Meta-analyses were conducted using STATA 15.0 software. The PCR, CCR, and MPR and the incidence of TRAEs with their 95% confidence interval (CIs) were evaluated for the studies included in the meta-analysis. Heterogeneity was assessed by the *χ*² test on Cochrane’s Q statistic and quantified by *I*² values. *I*² >50% and a P-value<0.05 indicated significant heterogeneity, and the random-effects model was used. Otherwise, the fixed-effects model was used. We performed subgroup analyses based on the type of experimental design, intervention PD-1 agent, and disease type. Sensitivity analysis was used to evaluate the stability and reliability of the results by the one-by-one elimination method. Funnel plots were generated to assess publication bias, and according to Egger’s test, P>0.05 manifested that there was no publication bias in the study.

## Results

### Literature search and study characteristics

**Figure 1:**
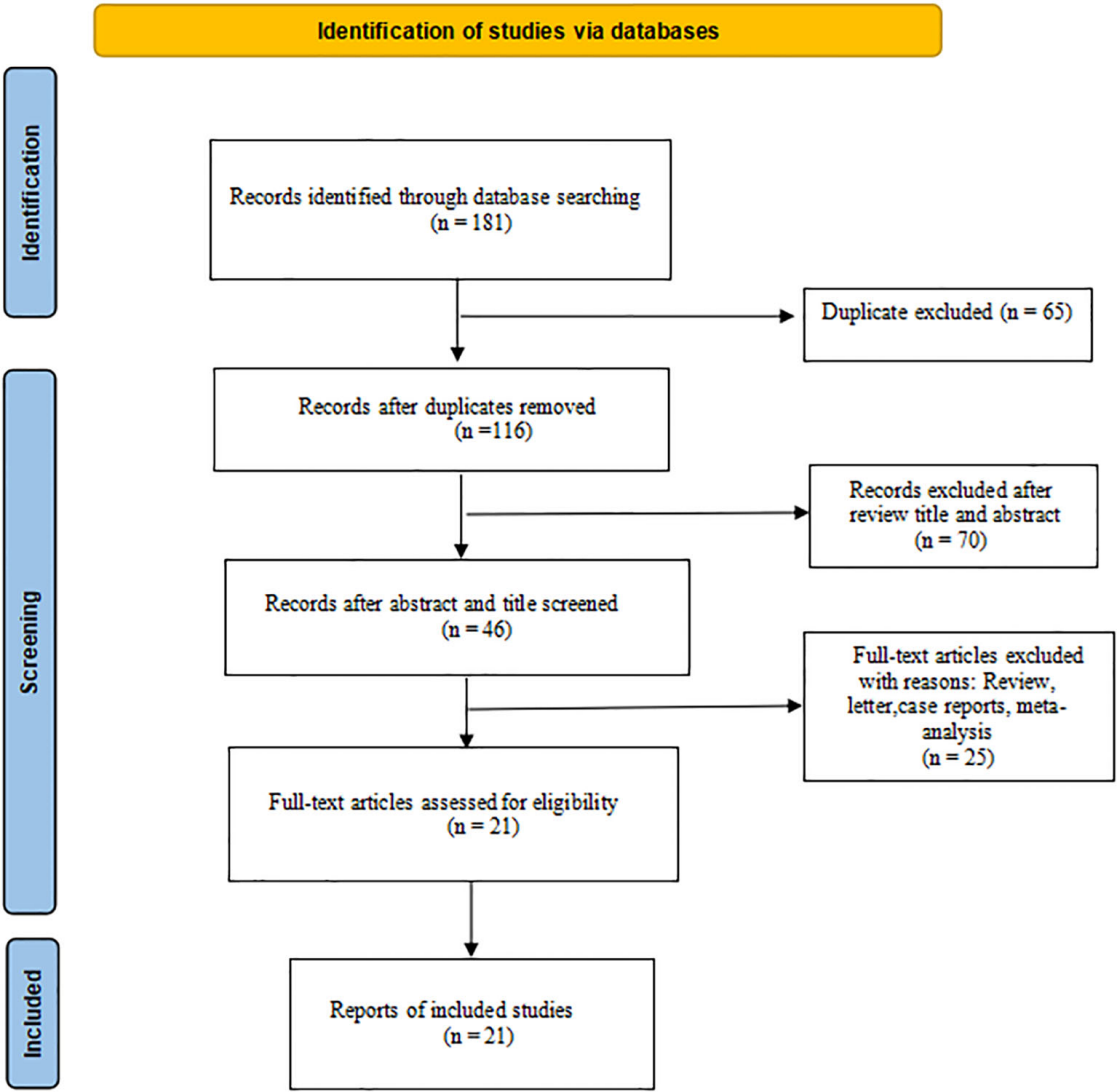
Flowchart of selection of included studies.

Initially, 181 types of literature were identified through searches of PubMed, Embase, and the Cochrane Library. Following the removal of duplicate studies, a total of 116 unique articles were included in the literature review. Subsequent screening of these articles resulted in 78 papers being assessed for potential inclusion. Further examination of the titles and abstracts led to the identification of 46 relevant records on the topic at hand; finally, 21 studies (7, 9, 14–32), including 803 patients, were identified for eligibility criteria in the survey via full-text review. The flowchart of the literature search procession is reported in **Figure 1**.

We incorporated studies spanning a 3-year period from 2021 to 2023. Within the selected literature, there were 17 cases of RC, encompassing 619 patients, and 4 cases of CRC, involving 184 patients. The reviewed studies comprised 12 prospective clinical trials and eight retrospective studies. The PD-1 inhibitors documented in the literature encompassed sintilimab, toripalimab, camrelizumab, dostarlimab, avelumab, pembrolizumab, tislelizumab, nivolumab, vudalimab, and bevacizumab. The detailed information is shown in **Table 1**.

**Table 1:** The basic characteristic of included studies of meta-analysis.

Author	Year	Diease	Number	F/M	Age(years)	Clinical stage TNM	Types of Clinical Research	Treatment cycle	PD-1 inhibitor	Median follow-up(months)	PCR	CCR	MPR
Zhou^32^	2022	RC	23	9/14	55(38-75)	T1-3aN0-1M0	prospective	4	Sintilimab	NR	2/23	10/23	6/10
Fox^16^	2023	CRC	38	11/27	NR	T1-4N0-+	retrospective	8	Sembrolizumab/Nivolumab/Vudalimab	28.2	11/18	NA	NA
Hu^7a^	2021	CRC	34	11/23	49(41-58)	T3-4N+	prospective	6	Toripalimab	14.9	15/17	NA	NA
Hu^7b^	2021	CRC	34	11/23	49(41-58)	T3-4N+	prospective	6	Toripalimab	14.9	11/17	NA	NA
Huang^17^	2022	CRC	18	NR	NR	NR	NR	NR	Sintilimab/Bevacizumab	4.5(1.5-9.4)	14/18	NA	14/18
Chen^14^	2023	RC	17	6/11	50(35-59)	T1-4N0-+	retrospective	4	Sintilimab	17.2(8.2-28.5)	3/16	9/16	NA
Li^18^	2021	RC	24	9/15	65(47-78)	T3-4N1N2aN2b	retrospective	NR	Sintilimab	11(6-24)	6/20	NA	4/20
Lin^19^	2021	RC	29	NR	57(31-73)	T3-4 N0 M0 or T1-4 N+ M0	retrospective	NR	Camrelizumab	NR	6/10	NA	NA
Liu^20^	2022	CRC	94	46/48	58	T3-4N0-N+	retrospective	4 (1⁃10)	NR	32(1-46)	39/94	NA	57/94
Lumish^21^	2022	RC	13	10/3	52(26-78)	NR	prospective	NR	Dostarlimab	NR	NA	7/13	NA
Michael^22^	2023	RC	33	10/23	55(31-76)	T3b-4N1-2M0	prospective	4	Avelumab	NR	NA	NA	NA
Zhang^23^	2022	RC	30	13/17	(57±16)	T3-4/N0-2/M0	prospective	NR	Camrelizumab	20(20-29)	13/27	7/30	NA
Salvatore^24^	2021	RC	101	62/39	63(23-82)	T3-4N+	prospective	6	Avelumab	NR	22/96	NA	59/96
Shamseddine^15^	2021	RC	40	14/26	58.5(31-74)	NR	prospective	6	Avelumab	13.2(3.6-31.9)	15/40	NA	27/40
Wang^25^	2021	RC	10	6/4	48(19-62)	NR	retrospective	5 (3-7)	NR	11.6(6.4-26.0)	NA	NA	NA
Wang^26^	2023	RC	62	NR	53(27-69)	NR	prospective	NR	Toripalimab	8(3-14)	18/32	29/62	37/59
Wang^27^	2023	RC	104	NR	55	T3-4N+M0	prospective	NR	NR	NR	29/59	29/104	NA
Wu^28^	2023	RC	25	NR	NR	NR	prospective	3	Camrelizumab	NR	17/21	NA	NA
Xie^29^	2023	RC	13	5/8	47(31-74)	T3-4N+	prospective	NR	NR	NR	3/13	10/13	NA
Yang^30^	2023	RC	20	7/13	55(23-74)	T3–4/N0–2/M0	retrospective	6 (4–10)	Pembrolizumab/ Sintilimab/Tislelizumab	NR	11/13	3/20	NA
Yang^31^	2022	RC	43	NR	63(32-77)	NR	prospective	3	Tislelizumab	NR	13/30	NA	NA
Zhang^9^	2022	RC	32	15/17	44(23-62)	T3~4N0~2M0	retrospective	6(4~10)	Pembrolizumab/ Sintilimab/Tislelizumab	14(3-28)	22/29	3/32	25/29

NA, not available; F/M, female/male; RC, rectal cancer; CRC, colorectal cancer; PCR, pathological complete response; CCR, clinical complete response; MPR, major pathological response; NR, not report.

### Risk-of-bias assessment

The NOS was employed to evaluate the quality of the included non-RCTs. There were 10 studies that received a score of eight points, whereas seven studies scored seven points, indicating a medium to high quality of the non-RCTs. For RCTs, the ROB tool was utilized for quality assessment in four studies. The most prevalent risks identified included the lack of blinding of participants and investigators, as well as the blinding of outcome assessment.

**Table 2** shows detailed information for each study.

Table 2The quality assessment of included studies of meta-analysis.

**Table:** 

RCT quality assessment						
Study	RANDOMISATION	ALLOCATION CONCEALMENT	BLINDING OF PARTICIPANTS AND INVESTIGATORS	BLINDING OF OUTCOME ASSESSMENT	SELECTIVE REPORT OF OUTCOMES	OTHER
Wang^27^	Low	Low	Unclear	Unclear	Low	Unclear
Wang^28^	Low	Unclear	Unclear	Unclear	Low	Low
Yang^30^	Unclear	Unclear	Unclear	Low	Low	Low
Hu^7^	Low	Low	Low	Unclear	Unclear	Unclear

**Table:** 

Non-RCT quality assessment										
Study	Selection (0–4)				Comparability (0–2)		Outcome (0–3)			Total
	REC	SNEC	AE	DO	SC	AF	AO	FU	AFU	
Chen^14^	1		1	1	1	1	1	1	1	8
Zhou^32^	1		1	1	1	1	1		1	7
Shamseddine^15^	1		1	1	1	1	1	1	1	8
Fox^16^	1		1	1	1	1	1	1	1	8
Huang^17^	1	1	1	1	1		1		1	7
Li^18^	1		1	1	1	1	1	1	1	8
Lin^19^	1	1	1	1	1		1		1	7
Liu^20^	1		1	1	1	1	1	1	1	8
Lumishl^21^	1		1	1	1	1	1		1	7
Michael^22^	1		1	1	1	1	1		1	7
Zhang^23^	1		1	1	1	1	1	1	1	8
Salvatore^24^	1	1	1	1	1	1	1		1	8
Wang^26^	1		1	1	1	1	1		1	7
Wu^28^	1	1	1	1	1	1	1		1	8
Xie^29^	1		1	1	1	1	1	1		7
Yang^31^	1	1	1	1	1		1	1	1	8
Zhang^9^	1		1	1	1	1	1	1	1	8

REC, representativeness of exposed cohort; SNEC, selection of nonexposed cohort; AE, ascertainment of exposure; DO, outcome not present at the start of the study; SC, control for important Factors; AF, additional factors; AO, assessment of outcome; FU, length of follow-up; AFU, adequacy of follow-up.

### Efficacy

**Figure 2:**
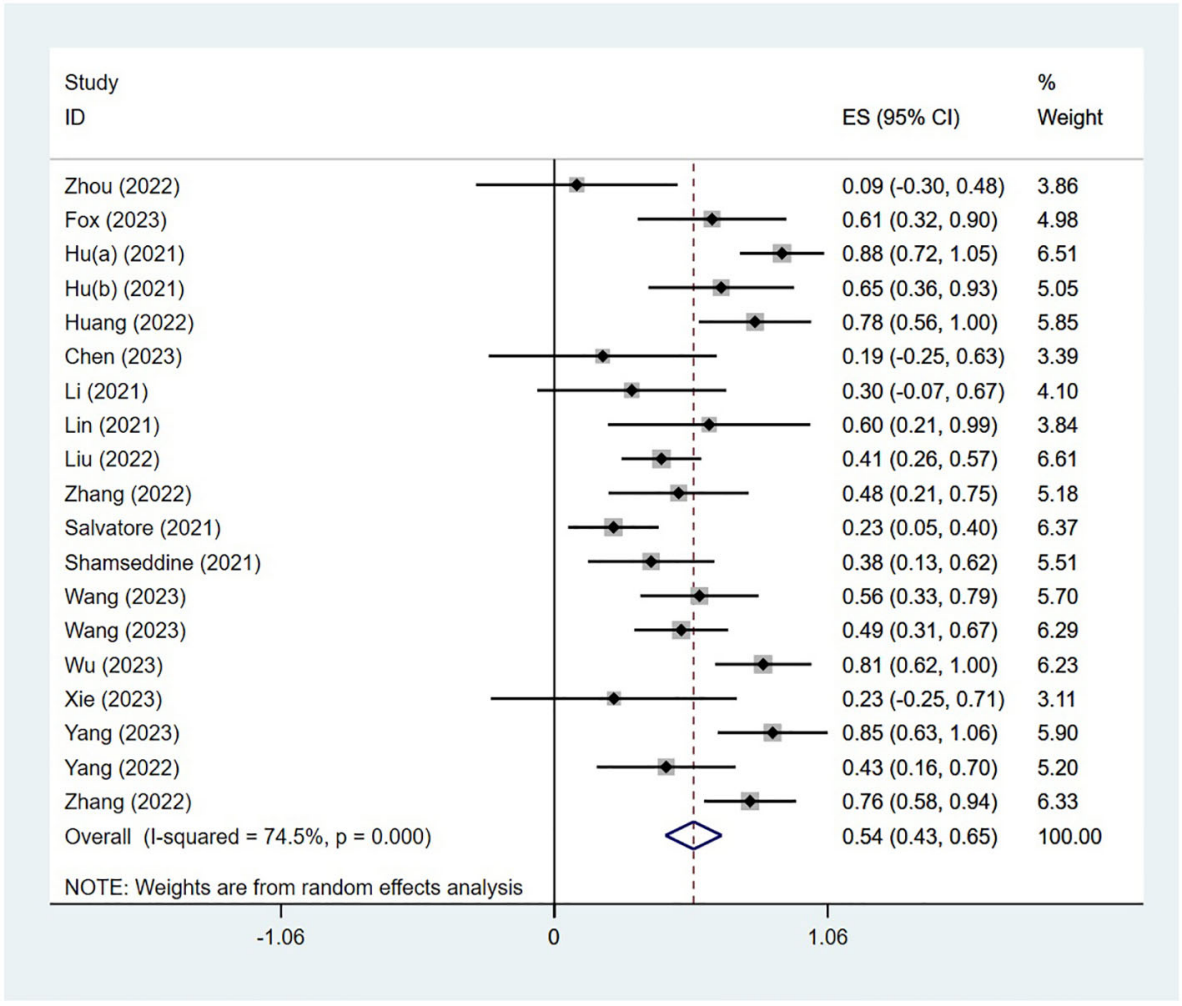
Forest plot of a meta-analysis of the pathological complete response rate.

The PCR was reported in 18 studies, and the PCR of PD-1 inhibitors in neoadjuvant treatment of LACRC was 54% (95% CI: 43%–65%, P<0.05). The forest plot of PCR is shown in **Figure 2**. The CCR was reported in nine studies, and the CCR was 40% (95% CI: 26%–54%, P<0.05). The MPR was reported in eight studies, and the MPR was 66% (95% CI: 56%–76%, P<0.05). The results are shown in **Table 3**.

### Subgroup analysis

We conducted subgroup analysis from three aspects: experimental design, PD-1 inhibitor interventions, and disease types. The identified studies included 12 prospective clinical trials (13–33), and the pooled PCR of PD-1 inhibitors in the prospective clinical trials of LACRC was 49% (95% CI: 32%–66%, P<0.05); the rate of PCR in eight retrospective studies was 57% (95% CI: 42%–72%, P<0.05).

The patients receiving sintilimab had a PCR rate of 55%. The patients who were administered toripalimab as a monotherapy exhibited a PCR of 73%, whereas those receiving camrelizumab demonstrated a PCR rate of 66%. Patients treated with avelumab displayed a PCR of 28%, those receiving Pembrolizumab had a PCR rate of 79%, and individuals administered tislelizumab had a PCR rate of 70%. In contrast, patients receiving pembrolizumab showed a CCR rate of 11%, whereas those treated with tislelizumab had a CCR rate of 17%. Additionally, patients receiving avelumab had an MPR rate of 64%. Among the 803 patients enrolled in the study, 619 (77%) were diagnosed with rectal cancer (RC), exhibiting PCR and MPR rates of 49% and 65%, respectively. The remaining 184 patients (23%) were diagnosed with CRC, with both PCR and MPR rates recorded at 67%. The detailed results of the subgroup analysis are shown in **Table 3**.

**Table 3:** The outcome of pooled PCR, CCR, MPR, and irAEs of meta-analysis.

Overall	Effect	95%CI	Heterogeneity test	
			I^2^(%)	P
**PCR**	0.54	0.43-0.65	74.5	<0.05
Subgroup analysis(design)				
prospective	0.49	0.32-0.66	80.9	<0.05
retrospective	0.57	0.42-0.72	64.2	<0.05
Subgroup analysis(PD-1 inhibitor)				
Sin	0.55	0.32-0.78	76.8	0.001
Tor	0.73	0.42-1.05	79.9	0.026
Cam	0.66	0.43-0.88	50.5	0.133
Ave	0.28	0.14-0.42	0	0.343
Pem	0.79	0.66-0.93	0	0.538
Tis	0.70	0.48-0.91	66.0	0.053
Subgroup analysis(disease types)				
RC	0.49	0.36-0.62	72.6	<0.05
CRC	0.67	0.47-0.87	77.8	<0.05
***CCR***	0.40	0.26-0.54	54.9	<0.05
*Subgroup analysis(design)*				
*prospective*	0.43	0.26-0.61	64.8	0.023
*retrospective*	0.34	0.09-0.59	49.2	0.116
*Subgroup analysis(PD-1 inhibitor)*				
*Sin*	0.32	0.10-0.55	41.8	0.161
*Per*	0.11	0.02~0.20	0	>0.05
*Tis*	0.17	0.02~0.20	0	>0.05
**MPR**	0.66	0.56-0.76	57.2	<0.05
Subgroup analysis(design)				
prospective	0.63	0.55-0.71	0.0	0.954
retrospective	0.61	0.34-0.89	85.6	0.001
Subgroup analysis(PD-1 inhibitor)				
Sin	0.66	0.42-0.90	71.8	0.014
Ave	0.64	0.53-0.74	0	0.583
Subgroup analysis(design)				
RC	0.65	0.52-0.78	65.3	0.013
CRC	0.67	0.51-0.83	43.7	0.183
***TRAEs***	0.27	0.17-0.37	55.2	0.004

### Safety

**Figure 3:**
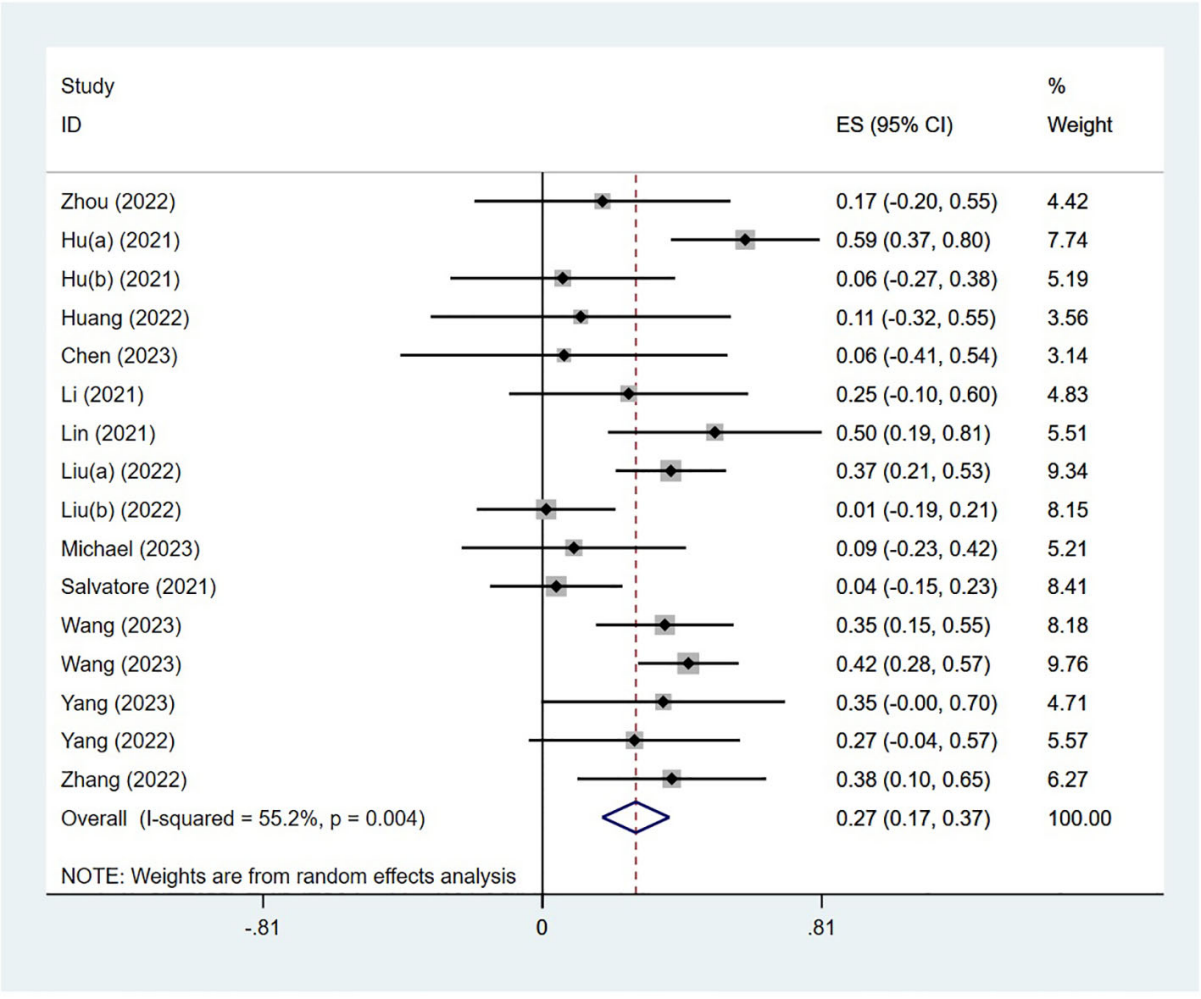
Forest plot of a meta-analysis of the irAEs rate.

The adverse effects were reported in 14 studies. The incidence of AEs was 27% (95% CI: 17%–37%, P<0.05). The forest plot of AEs is shown in **Figure 3**. The occurrence of grade 1–2 adverse events was 35.0% (95% CI: 19.7%–50.3%), whereas the incidence of grade 3–4 adverse events was 20.5% (95% CI: 7.8%–33.2%). The most common treatment-related AEs were mainly gastrointestinal reactions and skin adverse reactions, including fatigue, diarrhea, pruritus, nausea, pyrexia, abdominal pain, decreased appetite, bowel obstruction, hyperthyroidism, increased ALT, increased AST, thrombocytopenia, pneumonia, and neutropenia. Most grade 1–2 adverse effects can be improved through symptomatic treatment, and patients can continue to take medication such as vomiting. Only a small number of grade 3–4 treatment-related AEs have the potential to lead patients to discontinue the drug, indicating that the neoadjuvant regimen involving single-agent PD-1 inhibitors was generally deemed safe.

### Disease-free survival and overall survival

One multiple-center, cohort study including 20 patients reported (30) that the 2-year disease-free survival and overall survival in each group was 100%.

### Publication bias analysis and sensitivity analysis

**Figure 4:**
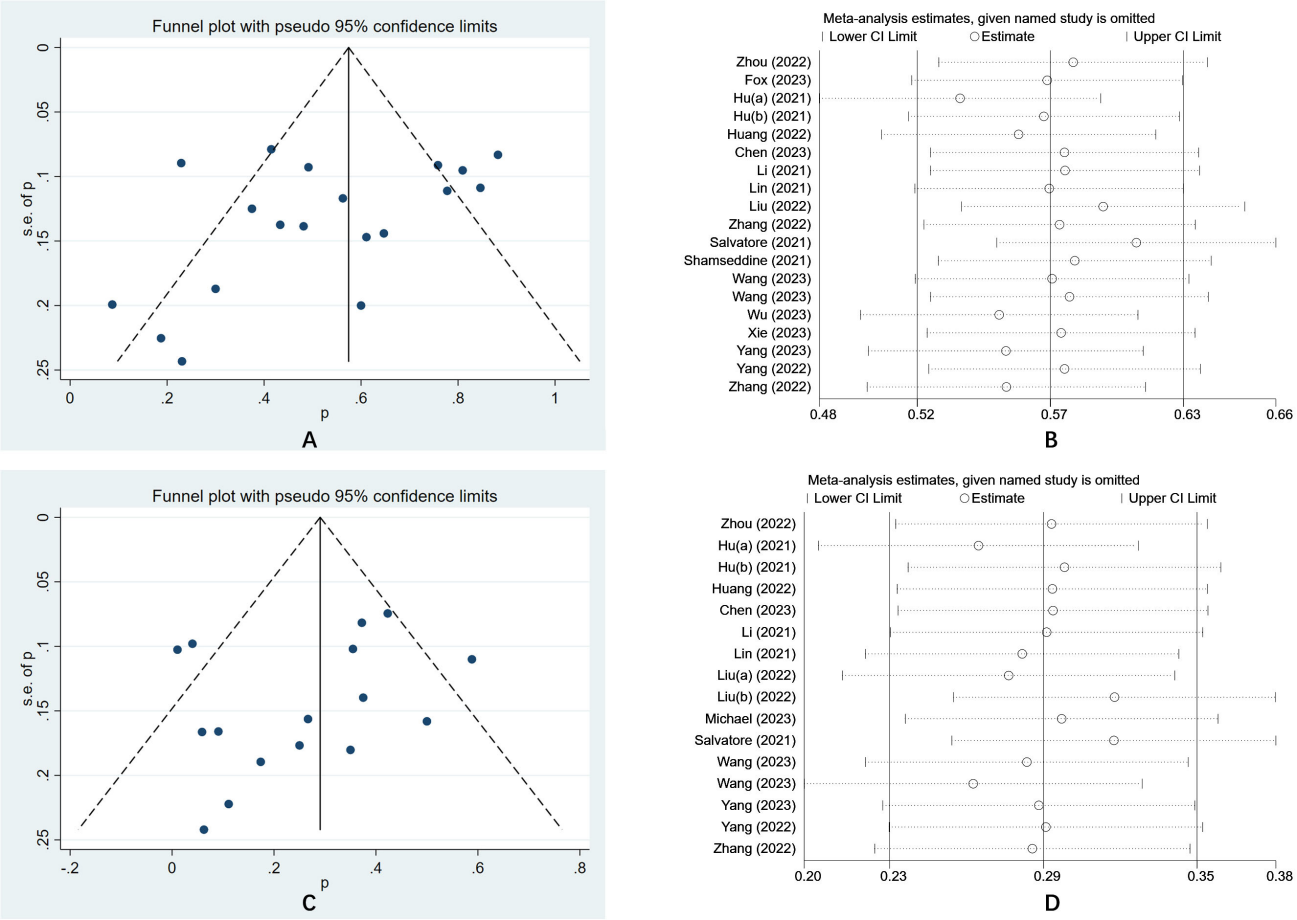
**(A)** The funnel plot of a meta-analysis of the PCR; **(B)** The sensitivity analysis of a meta-analysis of the PCR; **(C)** The funnel plot of a meta-analysis of the irAEs; **(D)** The funnel plot of a meta-analysis of the irAEs.

The funnel plot was a traditional method used to assess the presence of publication bias in meta-analyses, whereas Egger’s test provides a quantitative evaluation of such bias. In this meta-analysis, the results of Egger’s test (P = 0.152) and Begg’s test (P = 0.274) indicated an absence of publication bias in the included studies. Furthermore, the sensitivity analysis demonstrated that the findings of the study were generally stable. Refer to **Figure 4** for further details.

## Discussion

The objective of our meta-analysis was to examine the efficacy and safety of neoadjuvant monotherapy with PD-1 blockade in individuals diagnosed with LACRC. Efficacy was assessed using PCR, resulting in a 54% PCR, whereas safety was evaluated based on AEs, yielding a 27% AE rate.

Neoadjuvant therapy presents several benefits, including increased radical resection rates, decreased local recurrence rates, reduced tumor regression, and improved quality of life (33). However, the efficacy of neoadjuvant therapy in treating dMMR/MSI-H LACRC remains limited (34). Recently, ICIs targeting the PD-1/PD-L1 pathway have provided a new treatment option for malignant tumors. Neoadjuvant therapy can specifically bind to PD-1 or PD-L1 to block the PD-1/PD-L1 signaling pathway so that T cells can restore the immune response against tumors, thereby increasing the killing of tumor cells (35, 36). PCR was defined as tumors without any viable tumor cells in the resected primary tumor sample and all sampled regional lymph nodes. Recent results of clinical trials indicated that neoadjuvant therapy based on ICIs holds great potential in the treatment of LACRC. However, the current studies were limited by small sample sizes. To obtain more reliable results, we synthesized the published articles using a meta-analysis approach. This meta-analysis conducted in the present study revealed a pooled PCR rate of 54%. Previous studies have reported that the range of PCR rates was very large. For instance, the Ave-rectal trial (22) performed SCRT followed by six cycles of mFOLFOX6 and avelumab and found that 37.5% of the patients achieved PCR. The ANAVA study (37) used six cycles of avelumab from the beginning of nCRT and reached a PCR of 23%. While another study demonstrated a PCR rate of 48.1% (13/27) (19). Furthermore, in the study (NCT04304209) conducted by Gong Chen (14), four cycles of neoadjuvant sintilimab therapy were performed, and the PCR rate was 50% in patients with LACRC. In the PICC study, six cycles of neoadjuvant toripalimab with or without celecoxib resulted in high PCRs (65% and 88%) in patients (38). In reality, the PCR rate of neoadjuvant immunotherapy varies, but through our research, the overall PCR rate was approximately 54%. However, the current sample size was still relatively small, and further verification was needed.

Moreover, because the efficacy of various PD-1 blockade drugs may vary, resulting in differential PCR rates, we performed a subgroup analysis stratified by specific drugs. Within this analysis, patients treated with pembrolizumab, toripalimab, and tislelizumab exhibited higher PCR rates of 79%, 73%, and 70%, respectively. However, the current number of included studies is still relatively small, and further validation is needed. Our research findings indicate that neoadjuvant immunotherapy has yielded benefits for certain patients by enhancing PCR rates. However, a subset of patients did not experience these benefits. Future efforts should focus on refining patient selection criteria to identify those most likely to benefit, thereby advancing the objectives of precision medicine.

Furthermore, we evaluated the safety profile of PD-1 blockade by analyzing AEs and identified an incidence rate of 27%. Previous studies reported that the adverse reactions of neoadjuvant immunotherapy were diverse. For example, the common treatment-emergent AEs of any grade were leukopenia, reactive cutaneous capillary endothelial proliferation, and radiation proctitis (19). The common grade 3–4 treatment-emergent AEs were neutropenia and anemia (19). The ANAVA study (23) incorporated six cycles of avelumab commencing at the initiation of CRT. Among the 96 patients who were eligible for pathological assessment, 22 individuals (23%) attained a PCR, whereas 59 patients (61.5%) exhibited pathological regression. Notably, the incidence of grade 3–4 immune-related toxicities was limited to 4% (37). The incidence of adverse effects observed in the aforementioned clinical trials was comparatively low, particularly concerning immune-related adverse effects, which instilled considerable confidence in the outcomes (26, 37).

However, it is important to note that this study was subject to several limitations. Firstly, the sample sizes of the included studies were relatively small, warranting larger sample sizes randomized controlled studies in this area. Furthermore, there was presently a lack of direct comparisons regarding the efficacy of two distinct neoadjuvant immunotherapy groups, rendering head-to-head comparisons unfeasible. Consequently, this article employed a meta-analysis of rates. We anticipated the publication of additional clinical studies in the future, which would enable us to derive more reliable conclusions through evidence-based medicine. Additionally, the majority of studies were conducted in a single center, highlighting the need for large-scale, multicenter prospective randomized controlled trials to validate the long-term efficacy and safety of the treatment approach. Moreover, variations in neoadjuvant treatments among patients may have influenced the results, underscoring the necessity for the exploration of more effective treatment regimens tailored to individual patients. Furthermore, the majority of the articles had a limited follow-up period and did not provide data on progression-free survival and overall survival. Only one article presented relevant data in this regard. There is a need for future studies to report on long-term survival outcomes.

## Conclusion

The initial findings indicated that the neoadjuvant therapy utilizing the PD-1 inhibitor showed promise in terms of effectiveness and safety for LACRC patients.

## Data Availability

The original contributions presented in the study are included in the article/supplementary material. Further inquiries can be directed to the corresponding author.
